# Supplementary material for: Mitochondrial Dysfunction in Advanced Maternal Aged Cumulus Cells: A Possible Link to ATP Synthase Impairment?
Source: Biomolecules. 2024 Feb 26;14(3):281. doi: 10.3390/biom14030281 (PMC10967744; doi:10.3390/biom14030281)
Supplement: Supplementary file 1 [file biomolecules-14-00281-s001.zip › biomolecules-2841213-supplementary.pdf]

Supplementary data

Supplementary Table S1 - Controlled ovarian stimulation (COS) protocols used for women undergoing in vitro fertilization in the study.

COH protocols	*Number of patients*
Short protocol with antagonist	n=172
Short protocol with agonist	n=3
Long protocol	n=41
Mild stimulation	n=9

Note: *In all of protocols, hCG was injected when follicles reached 18 mm. 36 h after hCG injection, oocytes were retrieved transvaginally under ultrasound guidance*

Supplementary Table S2 - Sequence of primers used for RT-PCR study.

Primer name	Primer sequence (5'- 3')
**mtMinArc f**	GACATCTGGTTCCTACTTCAGGG
**mtMinArc r**	AGAGCTCCCGTGAGTGGTTA
**β2m f**	GGATTGGTATCTGAGGCTAGTAGG
**β2m r**	GAAATGAGGCTGGCAGAATAGG

Supplementary Table S3 - Semen parameters of cycles in the young and the advanced age groups

Semen quality parameters	***Young group***	***Advanced maternal age group***	***p-value***	*****
Age (years)	33.99 ± 5.04	38.86 ± 4.67	<0.0001	***
Volume (mL)	2.93 ± 1.68	2.82 ± 1.78	0.479	NS
Concentration (million/mL)	22.42 ± 27.41	33.09 ± 33.53	0.0155	*
Progressive motility (%)	30.14 ± 20.29	27.82 ± 17.58	0.549	NS
After 30 minutes of swim up				
Concentration (million/mL)	11.52 ± 22.97	15.05 ± 19.03	0.0313	*
Motility (%)	90.44 ± 28.34	93.53 ± 23.20	0.3866	NS

Note: *Data are presented as the mean ± standard deviation (SD) or number/%. Mann Whitney test was applied and p<0.05 was considered statistically significant.*

A

*Table 1 - **Clinical characteristics of patients in control group and young group***

Characteristics	*Control group*	*Young group*	*p-value*	*
**Number of patients**	40	140		
Age (y)	27.80 ± 4.61	29.74 ± 4.12	0.0096	**
BMI (Kg/m²)	22.31 ± 3.70	23.73 ± 4.24	0.068	NS
Basal serum AMH (ng/ml)	9.93 ± 2.71	4.29 ± 3.22	0.530	NS
Opu egg number	16.37 ± 10.14	14.29 ± 8.70	0.278	NS
MII oocyte number	10.19 ± 6.75	8.84 ± 5.70	0.356	NS

Note: Control group are young women without known infertility (that undergone fertility preservation or oocyte donation). Data are presented as the mean ± standard deviation (SD) or number/%. P<0.05 was considered statistically significant. AMH (anti-mullerian hormone) BMI (body mass index); CP (clinical pregnancy); D3 (Day 3); MII (metaphase II); OPU (ovum pick-up).

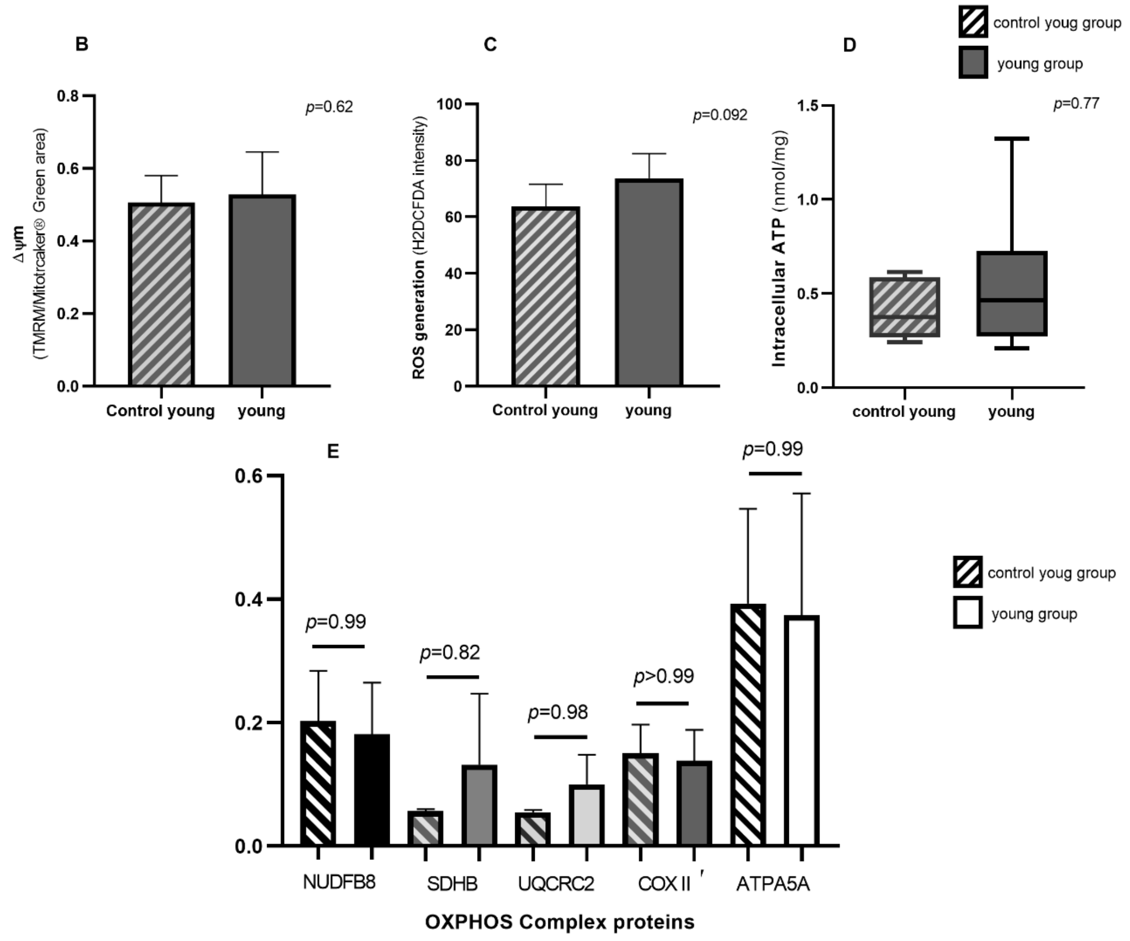

Supplemental Figure S1 – Reproductive potential is similar in control young group and young group. Clinical outcomes are similar between groups although control group is younger comparatively with young group (27.80 ± 4.61 versus 29.74 ± 4.12; p=0.0096), BMI (p=0.068), AMH levels (p=0.530), number of recovered (p=0.278) and MII oocytes (p=0.356) are similar. Mitochondrial function in control group and young group is equal, no differences were observed in ΔΨm between groups (p=0.62), neither in cytosolic ROS production (p=0.092) and intracellular ATP generation (p=0.77). The level of OXPHOS subunits protein is equal in both groups [NDUFB8 (p=0.99), SDHB (p=0.82), UQCRC2 (p=0.98) and COX2 (p>0.99) and ATP5A (p=0.99) subunits]. Mann-Whitney test was used for ROS and ATP production and one-way ANOVA for OXPHOS proteins, statistical significance considered when * $p < 0.05$, ** $p < 0.01$, and *** $p < 0.001$.
